# Rituxan-Induced Tumor Lysis Syndrome in a Patient With Diffuse Large B-Cell Lymphoma

**DOI:** 10.7759/cureus.16921

**Published:** 2021-08-05

**Authors:** Saffa Iftikhar, Waleed Khokher, Joan Gekonde, Nithin Kesireddy, Prabath Mudiyanselage

**Affiliations:** 1 Internal Medicine, University of Toledo, Toledo, USA; 2 Internal Medicine, The University of Toledo College of Medicine and Life Sciences, Toledo, USA

**Keywords:** large b cell lymphoma, electrolyte disturbances, rituximab, rituxan, tumor-lysis syndrome

## Abstract

Rituximab or Rituxan is a common drug used in the treatment of lymphomas. It is almost always used in conjunction with other chemotherapy regimens. Mechanism of action involves killing of the CD 20+ cells. Rituximab is implicated in precipitating tumor lysis syndrome (TLS) in patients with diffused large B cell lymphoma (DLBCL). The precise pathophysiological mechanism is not well known. Here, we present a case where the patient only received Rituxan for her newly diagnosed B-cell lymphoma which triggered a tumor lysis cascade. This in turn resulted in multiple electrolyte disturbances, multi-organ failure, and mortality. This report discusses the case presentation in addition to the different types of TLS and how this knowledge can be applied in the clinical setting for the future.

## Introduction

Rituximab is an anti-CD 20 monoclonal antibody. This is an essential drug used in treating B-cell malignancies. It works as both complement-mediated cytotoxicity and antibody-dependent cell-mediated cytotoxicity. It also uses indirect mechanisms involving apoptosis, structural changes and making cancer cells sensitive to chemotherapy. There are instances in which patients have shown resistance to the rituximab. As postulated by Cerny et al., the precise mechanism of action for resistance is not known. Studies have identified it as resistance involving anti-complement inhibitors CD55 and CD59 [1]. Here we report another case of rituximab-induced acute tumor lysis syndrome (ATLS). Tumor lysis syndrome (TLS) is a phenomenon that involves lysis of tumor cells, releasing their contents into the blood stream. This results in severe electrolyte abnormalities.

## Case presentation

A 49-year-old female with a past medical history significant for systemic lupus erythematosus (SLE), bipolar disorder, and chronic thrombocytopenia presented to the emergency department (ED) with a chief complaint of menorrhagia and dizziness. She also endorsed passage of multiple blood clots. She had a recent admission a month prior to this presentation where she received intravenous immunoglobulins (IVIG) for treatment of suspected immune thrombocytopenic purpura (ITP), intravenous (IV) steroids, and platelet transfusion. The patient was discharged at the time on a steroid taper of which she was taking prednisone 60 mg. Her platelet count on admission was 170*10^9^/L. She had a pelvic ultrasound done which was normal. Obstetrics-gynecology (OB-GYN) team started the patient on 5 mg Aygestin, orally twice daily for seven days.

In terms of the new-onset thrombocytopenia, she followed up with hematology-oncology a week prior to presenting to the hospital. Bone marrow (BM) biopsy and complete blood count (CBC) were reviewed. Initial BM biopsy was negative determined by flow cytometry with increased fibrosis and no lymphoma. She had a rapid decline in her platelets and this could not be explained with myelofibrosis. There was no improvement in her counts even with full dose prednisone. A repeat BM biopsy was found to be highly suspicious of lymphoma.

During her hospitalization, the patient was placed on an aminocaproic acid (Amicar) infusion and Nplate. Rituxan was also started for ITP. Steroid taper was continued. Two hours after the Rituxan administration, she became severely hypoxic requiring intubation for respiratory failure. Her hemoglobin dropped from 7.9 g/dl to 5.5 g/dl. Stat blood transfusion was ordered. CT chest/abdomen/pelvis was ordered which subsequently came back negative for hemorrhage. She underwent massive transfusion protocol. Labwork revealed lactate of 23 mg/dl, phosphorous of 14.4 mg/dl, uric acid 20 mg/dl, potassium of 6.9 mEq/l. Patient was diagnosed with ATLS. Rasburicase was administered and nephrology was consulted. She was started on urgent hemodialysis which she did not tolerate secondary to agitation and was transitioned to continuous renal replacement therapy (CRRT). She decompensated and required four pressors. Despite being on CRRT, the patient's metabolic parameters worsened. She remained hyperkalemic with potassium of 6.8mEq/l with worsening lactic acidosis suggestive of poor perfusion. The patient subsequently developed transfusion-related acute lung injury. There was difficulty with ventilation secondary to the lung injury. Her TLS had complete resolution but hemolysis continued to take place. She did not have adequate BM to produce red blood cells and platelets. She was also not a candidate for chemotherapy due to her clinical state. After meeting with the family, the decision was made to terminally extubate the patient.

## Discussion

From the literature review, there have been a total of seven reported cases of Rituxan-induced ATLS. Of the seven, two were reported in diffuse large B-cell lymphoma. Five reported cases included Burkitt’s lymphoma, B-cell chronic lymphocytic leukaemia, high-grade non-Hodgkin’s lymphoma, chronic lymphocytic leukaemia, and post-transplant patients. Here we report an additional case of Rituxan-induced TLS in a patient with diffuse large B-cell lymphoma. 

TLS is most commonly seen in patients diagnosed with non-Hodgkin’s lymphoma or acute leukaemia. The pathophysiology mainly involves lysis of tumor cells that then go onto release their contents into the bloodstream. This can lead to life-threatening electrolyte abnormalities including hyperuricemia, hyperkalemia, hyperphosphatemia, and hypocalcemia. 

The accumulation of these can result in end-organ damage effects namely, acute renal failure, cardiac arrhythmias, seizures, and eventual demise secondary to multi-organ failure [2]. 

TLS can be classified based on the Cairo-Bishop index of severity. This system was divided into lab TLS (LTLS) and clinical TLS (CTLS). Criteria for LTLS is diagnosed based on the following lab values - uric acid ≥ 476 μ mol/L, potassium ≥ 6.0 mmol/L, phosphorous ≥ 2.1 mmol/L for children, and ≥ 1.45 mmol/L for adults or each parameter increased by 25%, and calcium ≤ 1.75 mmol/L or decreased by 25%. The presence of two or more laboratory values within three to seven days after initiating cytotoxicity is sufficient to diagnose LTLS. CTLS is diagnosed when one of the following symptoms occur when LTLS criteria are met: Variables involved are creatinine, presence of cardiac arrhythmias, and seizures [3,4].

Treatment of TLS entails prophylactically avoiding the precipitation of this in high-risk patients by early risk stratification. Prevention mainly revolves around risk. High-risk patients should be managed with hydration and prophylactic rasburicase. Intermediate-risk patients are treated with allopurinol, hydration, or rasburicase. For low-risk patients, close monitoring is recommended [5]. 

## Conclusions

The case presented was a low-risk case. Mostly the risk is assessed once the patient is started on the chemotherapy regimen. In this case, the patient was not started on treatment other than Rituxan. The patient met the criteria for LTLS. She was treated appropriately for her electrolyte disturbances and was started on dialysis for her acute renal failure. Despite these interventions, the patient died. Since this is a rare albeit life-threatening consequence of the use of the drug, providers should be aware of this potential effect regardless of its use in chemotherapy. 
